# Index to the Periodical Literature of Dental Science and Art

**Published:** 1886-11

**Authors:** 


INDEX TO THE PERIODICAL LITERATURE OF DENTAL SCIENCE AND ART.—As presented in the English language. By J. Taft, M. D., D. D. S. Publishers: P. Blakiston, Son & Co., Philadelphia, 1886. Price, $2.00.

This valuable contribution to dental literature is in the form of an octavo of 212 pages, and a perusal of it shows that it embraces a useful index to the periodical literature of dentistry, dental periodicals and authors. To all in search of the valuable contributions to dental science and art that have appeared from time to time, and there are few progressive practitioners who do not have occasion for such research, this work will prove a valuable and time-saving aid. This work furnishes a list of all journals published since 1839, with the editor's names, etc., and an alphabetical list of the subjects of all dental articles with the names of the authors and journals in which such appeared. It also gives an index of authors contributing to dental literature, and cannot fail to prove greatly useful. Its subject matter is divided into: Index to the periodical literature of dentistry; Index to dental periodicals; Index to authors of dental literature.

